# Capsicum Enemata in Opium Poisoning

**Published:** 1883-03-20

**Authors:** James G. Kiernan

**Affiliations:** Chicago, Illinois


### CAPSICUM ENEMATA IN OPIUM POISONING.

By Jas. G. Kiernan, M. D., Chicago, Ill.

Opium poisoning is a not unfrequent occurrence from all sorts of causes. An opium habitue takes an overdose. A betrayed woman gets laudanum to quiet a colic and proceeds to commit suicide. Children drink laudanum left in their reach by injudicious parents and nurses. From all these and allied causes, opium poisoning is often an applicant for medical treatment. Any means of treating it which is readily applicable is therefore always in order. In the suggestion of capsicum enemata I can claim originality, but not priority.

Dr. Charles H. Hughes was the first to use capsicum enemata in a case of opium poisoning. A patient had taken opium with suicidal intent, and Dr. Hughes being called in consultation by Drs. Rœmer, Hypes and others, after the usual routine remedies had been used, ordered an enema of one drachm each of aqua ammonia and tincture of capsicum, using coffee for a vehicle. The patient rapidly rallied and recovered.

During the year 1881 I was called to a case which gave the following history: A patient suffering from the insomnia of a prolonged debauch purchased two ounces of laudanum, one of which he swallowed. Within half an hour he had sunk into a deep slumber. A physician was then called who evacuated the stomach by means of the stomach-pump, relieving the patient of about half the laudanum taken.

This physician found that despite the use of strong coffee and constant movement the patient did not improve. Dr. J. S. Jewell was then called in consultation who advised the use of atropine. Under all these varied means of treatment there were temporary rallies, but after six hours of constant treatment the patient seemed to sink into and remain in a very deep coma. At this stage of affairs I was called in consultation, and having some faith in the old idea of a derivative action, ordered three drachms of tincture of capsicum to be poured directly into the rectum. The effect was almost magical. The patient walked around rather briskly, talked freely, and in about an hour was in his usual condition, other than being much exhausted and complaining of great dryness of the throat, obviously the result of the atropine.

In a second case a five year-old child obtained possession of a bottle of laudanum belonging to its father who was a victim of gastric cancer, and in consequence an opium habitue. From the bottle the child drank approximatively about a teaspoonful. Atropine, emetics, the stomach-pump and the galvanic battery were tried with temporary success. But the influence of the laudanum manifested itself in a gradually increasing coma. Remembering my former experience I ordered an equal quantity of tincture of capsicum to be poured into the rectum. The result was a slower but equally permanent success. The child, for some time after, suffered from inflammation of the rectum, from which it made a slow recovery. From the case narrated by Dr. Hughes, and the two just cited, it would seem that this measure would be at least a good addendum to other means of treatment. Dr. Hughes claims to have had equally goo.l results from capsicum enemata in chloral poisoning. Hypodermic injections of strychnia being used in addition.—*Med. Weekly.*

